# Celebrating 350 years of the Royal Society

**DOI:** 10.1098/rsta.2009.0267

**Published:** 2010-03-13

**Authors:** Martin Rees

**Affiliations:** President of the Royal Society

Back in the 1660s, the Royal Society’s founders met regularly to discuss scientific ideas and perform experiments; the outcomes of their experiments and discussions were recorded in the Society’s Transactions. The world of science has been utterly transformed since that era. Nonetheless, these activities, pioneered by the Society, remain the accepted procedures whereby scientific ideas are criticized, refined and codified into publications that become part of ‘public knowledge’.

The papers in this special anniversary issue of the *Philosophical Transactions* address themes that, in some cases, could not have been conceived even 50 years ago. But their range reflects, nonetheless, a continuity and concordance with the Society’s earliest years. The ‘ingenious and curious gentlemen’ who established the Royal Society enjoyed speculation. But they were also intensely practical and engaged with the problems of their time: the rebuilding of London after the great fire, improvements to timekeeping, navigation and so forth. The papers here cover topics ranging from the remote parts of the Universe to literally down-to-earth issues of energy and material science.

Right from the start, the Society was networked internationally. That is, of course, even more the case today; and worldwide expertise is reflected in the distinguished authorship of the papers in this special issue. This special issue is a most welcome contribution to the range of activities and events whereby the Royal Society is marking its 350 years of support for science and scientists, and highlighting the importance of science in meeting the challenges of the twenty-first century.

